# Enhanced detection method for corneal protein identification using shotgun proteomics

**DOI:** 10.1186/1477-5956-7-23

**Published:** 2009-06-29

**Authors:** Mitchell L Meade, Pavel Shiyanov, John J Schlager

**Affiliations:** 1 Applied Biotechnology Branch, Biosciences and Protection Division, Human Effectiveness Directorate, 711th Human Performance Wing, Wright-Patterson AFB, Ohio, USA

## Abstract

**Background:**

The cornea is a specialized transparent connective tissue responsible for the majority of light refraction and image focus for the retina. There are three main layers of the cornea: the epithelium that is exposed and acts as a protective barrier for the eye, the center stroma consisting of parallel collagen fibrils that refract light, and the endothelium that is responsible for hydration of the cornea from the aqueous humor. Normal cornea is an immunologically privileged tissue devoid of blood vessels, but injury can produce a loss of these conditions causing invasion of other processes that degrade the homeostatic properties resulting in a decrease in the amount of light refracted onto the retina. Determining a measure and drift of phenotypic cornea state from normal to an injured or diseased state requires knowledge of the existing protein signature within the tissue. In the study of corneal proteins, proteomics procedures have typically involved the pulverization of the entire cornea prior to analysis. Separation of the epithelium and endothelium from the core stroma and performing separate shotgun proteomics using liquid chromatography/mass spectrometry results in identification of many more proteins than previously employed methods using complete pulverized cornea.

**Results:**

Rabbit corneas were purchased, the epithelium and endothelium regions were removed, proteins processed and separately analyzed using liquid chromatography/mass spectrometry. Proteins identified from separate layers were compared against results from complete corneal samples. Protein digests were separated using a six hour liquid chromatographic gradient and ion-trap mass spectrometry used for detection of eluted peptide fractions. The SEQUEST database search results were filtered to allow only proteins with match probabilities of equal or better than 10^-3 ^and peptides with a probability of 10^-2 ^or less with at least two unique peptides isolated within the run along with default Xcorr values. These parameters resulted in the identification of over 350 proteins, including over 225 new proteins not previously detected in the cornea by mass spectrometry. In addition, corneal layer separation resulted in identification of nearly every protein that was identified in the complete cornea assay. The epithelium and endothelium each revealed many unique proteomes specific to each layer. In the endothelium, the protein olfactomedin-like 3 was identified for the first time in the cornea by this analysis. Olfactomedin-3 is a neuronal expressed protein also known as optimedin that stimulates formation of cell adherent and cell-cell tight junctions and its expression modulates cytoskeleton organization and cell migration. However, the function of this protein in rabbit corneal endothelium is currently unknown.

**Conclusion:**

This manuscript presents a description of a more comprehensive proteomic profile for mammalian cornea compared to past methods. The use of simple dissection procedures of the tissue and the application of long chromatographic gradients, many more proteins can be identified.

## Background

The cornea is a transparent connective tissue that provides a majority of the refraction for the eye. In addition, the cornea also acts as a hydrated protective external barrier for the rest of the eye and provides clear optical components for image focusing on the retina. There are three main layers of the cornea: the epithelium, the center stroma, and the endothelium, with Descemet's membrane residing between the stroma and endothelium. The external epithelium the layer is 5–6 layers of stratified cells cumulatively ~50 μm thick. The epithelium provides the primary protective layer of the cornea and quickly regenerates new layers for maintenance of this external barrier function. The connective tissue derived stroma constitutes a majority of the mass and optical thickness of the cornea (~450 μm) and consists of parallel fibrils of collagen that provide optical clearness and the light refractivity of the cornea for image focus. The endothelium averages only one cell layer thick and is known to be physiologically responsible for assuring appropriate corneal hydration of the stroma by pumping fluid and nutrients in and out to the aqueous humor.

Previous proteomic investigations of the cornea have been performed on complete isolated cornea powder with such techniques as 2-D PAGE, 1-D PAGE and SCX fractionation of the extracted protein mixture prior to LC-MS/MS identification. [1,2] A study of human corneal proteins by Karring *et. al*. showed the identification of 141 unique proteins using 2-D electrophoresis followed by spot LC-MS/MS analysis. While all techniques require one to reduce the complexity of the sample prior to LC-MS/MS analysis, low abundance proteins may not appear on the gel or may elute throughout the SCX fractionation, thus not allowing the component to be present for detection by the mass spectrometer. [3] Front end sample preparation methods with procedure simplicity and low amounts of sample handling steps are the key to providing the most successful approach for detecting the greatest protein diversity within a biological sample.

Shotgun proteomics is a technique in which a complex mixture, such as a tissue in our study, is lysed, digested and separated by liquid chromatography on-line prior to MS/MS analysis. [4-7] Shotgun proteomics has the ability to identify many proteins from a complex mixture in a short period of time. The disadvantage of this approach is that the instrumentation may not be able to analyze every peptide as it elutes in a highly complex protein mixture. This problem can be overcome with the use of a longer chromatographic gradient afforded by ultrahigh pressure liquid chromatography (UPLC), which can reduce the rate of peptide elution entering the mass spectrometer and the complexity of subsequent analysis with minimal peak broadening, thus enabling the mass spectrometer and software to analyze cleanly more peptides. In this investigation, detection of corneal proteins was substantially enhanced by separating the epithelial and endothelial layers from the collageneous stroma layer. The extended chromatographic technique provides a larger dataset of proteins that are present in these layers and for the corneal tissue. In this paper we present a comparison of the protein sets identified in the epithelium, endothelium, and a complete pulverized cornea.

## Results

### Corneal protein identification by shotgun proteomics

**Figure 1:**
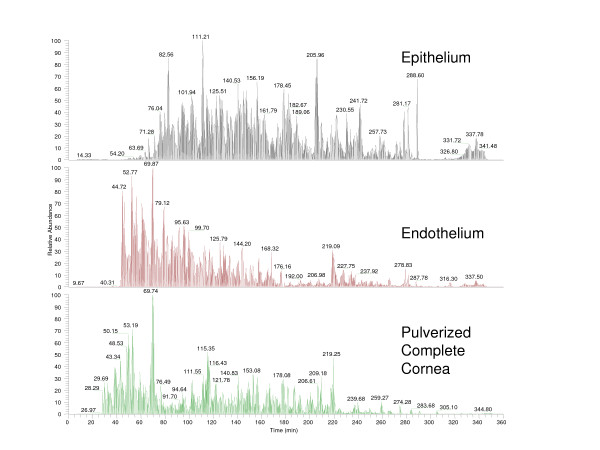
**Base peak chromatograms for the epithelium (top), endothelium (middle), and complete pulverized (bottom) corneal samples**.

Rabbit corneas were purchased and processed for subtissue proteomic analysis using LC/MS/MS shotgun proteomics. LC/MS/MS shotgun proteomic methods have the ability to probe the proteome of a complex mixture created from minimal sample preparation compared with other techniques. The separation of the corneal epithelium and endothelium from the vast amount of collagen present in the cornea, which dilutes lower abundance proteins, significantly enhanced the number of proteins that can be identified. In addition, the implementation of long chromatographic separation time results in a much larger number of MS/MS scans to be searched for assessment of unique peptide data. [8] Each protein fraction analysis produced approximately 30–35 k MS/MS spectra that were searched against the protein database. An example of base peak chromatograms from each fraction is shown in Figure 1. SEQUEST analysis of the MS/MS data of the epithelium identified 279 unique proteins while not counting isoforms or nearly identical homology of proteins. This includes 181 (65%) proteins not previously identified in the cornea by proteomic procedures. The list of proteins identified in each fraction is shown in the Additional File 1. This result compares to approximately 120 proteins that were identified using a three hour separation time with the same samples and instrument (Data not shown). The use of longer separations resulted in a greater than 250% increase in the number of protein identifications with minimal effect on the chromatographic resolution.

**Figure 2:**
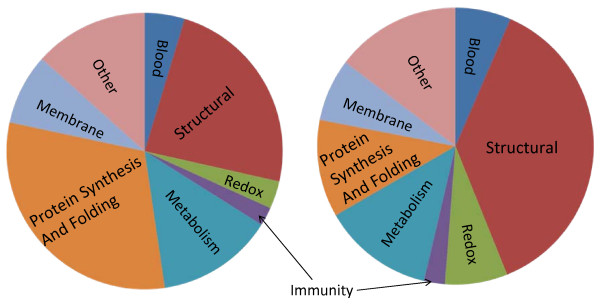
**Identified protein distribution by function in the epithelium (left) and endothelium (right)**.

Once identified, the proteins were grouped into: structural, protein synthesis and folding, redox state management, metabolism, immunity, membrane associated, blood and plasma proteins and other function. Figure 2 (left) shows the functional distribution of these proteins for the epithelium. There were 66 proteins (24%) identified in the epithelium with a structural role. These include alpha collagens, keratins, actin, tubulin and decorin among others. Collagens are localized in the basement membrane and the stroma and these proteins may be acquired as cross region contamination during removal of the individual layers. [9] Proteins involved in protein synthesis and folding were also abundant in the epithelial fraction with 85 proteins (31%) identified. The protein identities include specific histones, ribosomal proteins, heterogeneous ribonucleoproteins, along with heat shock proteins involved in protein folding. Since the epithelium of the cornea is exposed and responsible for protection of the eye, there were 7 (3%) immunity related proteins that were identified including immunoglobulins and beta defensin. There were 9 (3%) redox associated proteins that were identified including peroxiredoxins -1,-5 and -6 and glutathione-S-tranferase. Metabolic proteins accounted for 38 (14%) proteins that were identified in the epithelium. These identified proteins included many dehydrogenases, such as aldehyde dehydrogenase, lactate dehydrogenase and glyceraldehyde 3-phosphate dehydrogenase frequently found in large abundance in metabolically active structures and consistent with prior published data. [10] Other less abundant proteins, such as carbonyl reductase 1, a reducer of carbonyl containing compounds, and phospholipase C-alpha, a possible ER enzyme, were among the proteins that were identified. [11,12] Identified membrane proteins were involved in the import and export of molecules through the cell membrane, as well as, membrane structural proteins. Of the 22 proteins (8%) found as membrane associated proteins in the epithelium, many identified were desmosomal associated proteins. Desmosomes are known to be present in epithlelial tissues and are responsible for cell to cell adhesion. Desmosomal proteins identified included: junction plakoglobin, plakophilin-1, envoplakin, desmoglein among others. These proteins have been detected by indirect methods in the cornea, as well as in other epidermal tissues by LC/MS/MS, however this is the first direct detection of these corneal proteins by direct LC/MS/MS methods. [13,14] Although the cornea is not vascularized, there were 14 (5%) blood and plasma associated proteins that were identified. These included serum albumin, transferrin, hemoglobin subunits, as well as Annexin V and VIII. There were also 35 (17%) proteins of presently unknown or other function, which included the 14.3.3 proteins, and retinol binding protein.

**Figure 3:**
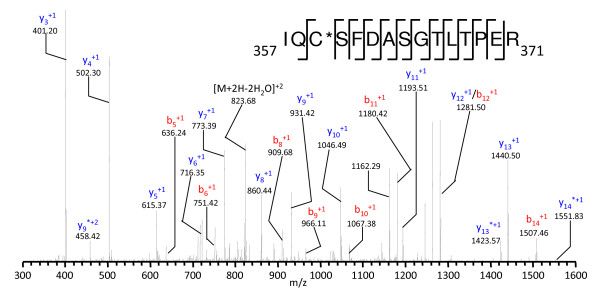
**MS/MS spectrum of the _357_IQCSFDASGTLTPER_371 _peptide from Olfactomedin-Like 3 in the corneal endothelium**.

In the endothelial fraction, 131 unique proteins were identified with SEQUEST, of which 60 proteins (46%) have not been previously detected in the cornea by mass spectrometry. The protein set in the endothelial fraction is markedly different than the set found for the epithelium. The lower number of overall protein identifications in this layer compared to the epithelium may be due to this layer being thin, one cell layer thick, and lower overall protein amounts. The breakdown of the functionality of these proteins is shown in Figure 2 (right). Structural proteins dominated the endothelial fraction with 44 (34%) proteins identified with the major constituents being intracellular keratins and actins and extracellular collagens IV, VI, VIII, XII, XVIII and perlecan. Collagen IV, XVIII and perlecan are associated with the cell adherence region in extracellular basement membrane. Although a significant amount of collagen proteins were identified in the endothelium, collagen IV is known as the major endothelial collagen. [15] Proteins known to interact with collagen IV such as Lipocalin-7, Fibulin-5, and nidogen were also identified. [16] None of these proteins were found to be present in the epithelial layer. The presence of the collagen VIII is most probably from isolation of Descemet's membrane components as it is a major protein in this layer. [17] There were 14 proteins (11%) identified involved in protein synthesis and folding. These identified proteins match those that were identified in the epithelial fraction. They include the histones, heterogeneous ribonucleoprotein A and eukaryotic translation initiation factor. There were 3 (3%) proteins involved in immune function from endothelium with immunoglobulins and clusterin identified. A total of 16 (12%) proteins were identified with a metabolic function in the cell. Major metabolic proteins in the cornea, such as glyceraldehyde-3-phosphate dehydrogenase, transketolase, and retinal dehydrogenase were identified with large sequence coverage for each. Apolipoprotein E, a cholesterol transport protein, and cathepsin F, a papain-like cysteine neuronal lysosomal protease of unknown function, were also identified in this group and found only in the endothelium. [18-20] Ten (7%) redox maintenance and antioxidant proteins were identified in the endothelium. The identified proteins are similar to those identified in the epithelium with glutathione-S-transferases and peroxiredoxins, while lysyl oxidase-like 3, a member of the protein family used to maintain and strengthen connective tissue collagen and elastin through protein cross-linking, was found only in the endothelium. [21,22] There were 12 membrane-associated proteins (9%) identified in the endothelium. These include the cell to cell adhesion proteins vitronectin, laminins, nephronectin and fibulin-1. [23,24] Eight blood and plasma associated proteins (12%) were identified in endothelium including cochlin, a trabecular meshwork protein, and hemopexin, indentified only in the endothelium. Finally, 18 proteins (14%) identified had an unknown or another function. An abundant endothelial protein indentified with an unknown function was olfactomedin-like 3 (OML-3), a glycoprotein thought to play a role in neuronal development where it has been identified previously in the trabecular meshwork of the eye. [25] However, there is no known literature suggesting a role for OML-3 in the corneal endothelium. 15 unique peptides were identified from OML-3, resulting in 30% sequence coverage. The MS/MS spectrum from the 357–371 peptide from this protein is shown in Figure 3. Another protein identified in the endothelium with an unknown corneal function was cartilage acidic protein 1 (CAP-1), although CAP-1 has been previously detected in genomic studies of the cornea[26]. Both of these proteins were identified in the pulverized cornea sample as well, although many less peptides were identified.

**Figure 4:**
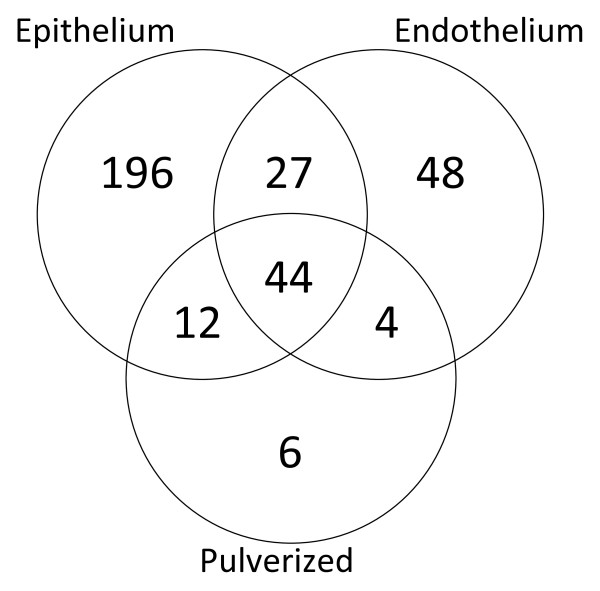
**Venn diagram of the proteins identified by SEQUEST in each of the three sample types**.

A total of 66 proteins were identified in the pulverized complete cornea sample. These proteins were mainly abundant proteins within the cornea such as keratins, collagens, and enolase. Nearly all proteins found in this fraction (60) were identified collectively in the other fractions. There were only 4 proteins identified in this sample that were found in the endothelium only, while 12 proteins were identified in this sample found only in the epithelium. Since the collagenous stroma is the largest layer of the cornea comprising approximately 70% of the total cornea, the comparatively lower abundant proteins in the epithelium and endothelium were less likely to be detected by the mass spectrometer. Figure 4 shows a Venn diagram of the number of proteins identified in each of the layers. This diagram illustrates the significant difference in the proteins identified in the epithelial and endothelial fractions. There were 196 proteins identified solely in the epithelial fraction, 48 proteins identified solely in the endothelial fraction and 27 found in both fractions while none of these proteins where identified in the pulverized cornea. With 60 (90%) of the 66 total whole corneal proteins identified from the pulverized cornea analysis, the method provides sufficient coverage with just analysis of epithelium and endothelium. Even more importantly, the separation of layers resulted in the identification of an additional 270 proteins versus the pulverizing of the entire cornea.

### Immunohistochemistry

**Figure 5:**
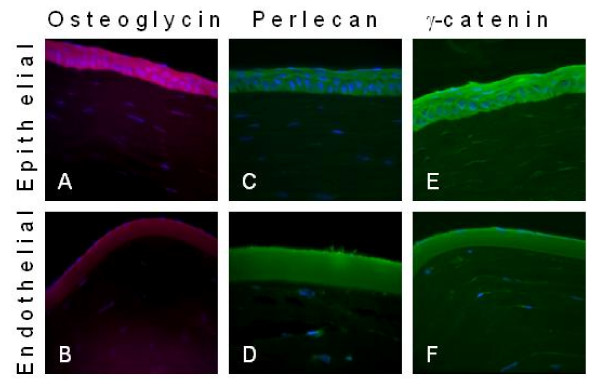
**Immunostaining of corneal slices for osteoglycin (A-B), perlecan (C, D) and γ-catenin (junction plakoglobin)(E, F)**.

To confirm the presence of junction plakoglobin (γ-catenin), osteoglycin, and perlecan, the corneas were sliced and probed with antibodies to osteoglycin, perlecan and junction plakoglobin. The images of the immunostaing from these three proteins are shown in Figure 5. The staining for osteoglycin showed that it is present in the epithelium and endothelium, but not in the stroma. Mass spectrometric data showed osteoglycin in the epithelium and endothelium portions, but no peptides were found in the complete pulverized cornea. Perlecan staining showed a majority of the protein resides in the endothelium, while the junction plakoglobin was found in the epithelium. Both of these findings are consistent with the mass spectrometry data as well as previously published immunostaining data. [27,28]

## Discussion

The cornea is a transparent tissue and the identification of more proteins by direct measurements can aid in future studies of corneal function. Our described LC/MS/MS shotgun proteomic method has the ability to probe the corneal tissue protein content beyond other techniques. The separation of the regions of the tissue and using a six hour liquid chromatography gradient were essential in probing the less abundant proteins. The use of a six hour separation gradient enabled the identification of twice the number of proteins compared to a three hour chromatographic separation (data not shown). The epithelium had the most proteins identified with 279, while the endothelium had 121 and the pulverized sample had 66 proteins identified. The epithelial and endothelial samples contained nearly every protein identified in the pulverized sample. The epithelium and endothelium also had many different proteins that were found in only one layer. As expected, many basement proteins, such as collagen IV, fibulins and fibronectin, among others were identified in the endothelium while the epithelium had many proteins identified as actively dividing tissue region, including eukaryotic translation and elongation factors, as well as, many ribosomal proteins.

Identified corneal proteins were found to be from all regions of the cell, including the extracellular matrix. Many proteins identified were responsible for the structural integrity of the cell including keratins, collagens and other proteins that interact with these proteins to form the cytoskeleton of the cell. Proteins involved in synthesis and folding comprised the largest number of identified proteins. This group includes proteins involved in the transcription, translation and maintenance of functional proteins in the cell. These proteins were found in all areas, but were significantly represented in the rapidly replenishing cellular epithelium layer. The epithelial fraction also contained many ribosomal protein subunits, while none were found in the other samples. Major metabolic proteins indentified included α-enolase and a number ofidentified dehydrogenases. Other proteins responsible for metabolism of carbohydrates and lipids such as pyruvate kinase to apolipoproteins, respectively, were also represented.

Another advantage to this method was that very large proteins such as membrane-associated proteins often lost in preparation for gel analysis were detected by the mass spectrometer. The membrane proteins detected were responsible for the passage of molecules through the membrane and extracellular adhesion molecules. The epithelium and endothelium had very different proteins that were identified with this function. Many of the epithelial membrane proteins were desmosomal proteins. Of the 32 total proteins identified, only 3 were found in both the epithelium and endothelium.

Immune-related proteins were found fairly homogeneously across all layers with the main proteins identified being immunoglobulins. Many other blood and plasma proteins identified were representative of major proteins found in blood. Serum albumin was by far the most abundant protein which is to be expected. Other major blood proteins such as transferrin and hemoglobin subunits were identified as well. These proteins may be associated with the tissue or could be associated from the excision of the tissues during isolation. Lastly, redox maintenance/balance proteins detected in the epithelium and endothelium were found with peroxiredoxins, gluthionone-S-transferases and superoxide dismustases identified in both layers.

In order to confirm and localize a number of proteins immunohistochemistry was employed and detected with a confocal fluorescent microscope. Three proteins were chosen and tissues stained for osteoglycin, perlecan and junction plakoglobin. Osteoglycin was identified in both the epithelium and endothelium by mass spectrometry and primary antibody staining show the presence of this protein in both regions. Perlecan is a major endothelial protein and was shown to be located mainly in the endothelium. However, there was some staining in the epithelium which corresponds to the mass spectral data that also identified the protein, although with only two unique peptides compared to over 50 unique peptides found in the endothelium. Junctional plakoglobin was found mainly in the epithelium, consistent with the mass spectrometric data. There was also junctional plakoglobin detected by immunohistochemistry in the endothelium although this protein was not identified with the mass spectrometer.

## Conclusion

Although corneal proteomics has been performed in the past, this paper presents detection of many new proteins by a direct method. This manuscript proves that the removal and separate identification of proteins of the epithelium and endothelium combined with longer (six hour), chromatographic gradients identified many new proteins. This LC/MS/MS shotgun proteomic method has the ability to probe the complex corneal protein mixture with minimal protein sample preparation compared to other techniques. A total of 341 unique proteins were identified in mature rabbit cornea using this method for the epithelial and endothelial layers. Of these, 227 proteins (67%) have not previously been identified in the cornea by mass spectrometry. The ability of a chromatographic system to separate peptides over a long gradient time was crucial for the identification of a greater number of proteins in any complex mixture. The data shows that the separation of the corneal layers results in many more proteins identified and retains identification of over 90% of the proteins (60 of 66) in pulverized whole cornea. As a larger database of rabbit proteins is assembled, we feel confident that more proteins will be identified. This paper shows an expanded rabbit corneal protein library that may be useful in further corneal proteomic or deferential phenotype physiology studies.

## Methods

### Corneal protein extraction

Mature rabbit corneas were purchased from Pelfreez Biological (Rogers, AK). Corneas were stored at -20°C until analysis. The connective tissue around the cornea was removed prior to protein extraction. The cornea was cut in half along the sagittal plane with surgical scissors and washed briefly in 50 mM Tris-HCl (pH8.0). The cornea was then blotted to remove residual liquid. The epithelium and endothelium were removed from the stroma by gentle scraping with a surgical razor blade. The epithelium and endothelium are not bound tightly to the collagenous stroma and are easily removed. The toughness of the stroma, due to collagen, aids in facilitating this removal with minimal cross contamination from the stromal layer. To prepare the pulverized corneal powder, the complete cornea was frozen in liquid nitrogen and pulverized by a Bio-pulverizer by Biospec (Bartlesville, OK). The epithelial, endothelial and pulverized tissues were placed in a lysis buffer containing: 50 mM Tris-HCl (pH = 8.0), 8 M urea, 100 mM NaCl, and 10 mM DTT. Each sample was sonicated for one minute in an ice bath. After sonication, samples were additionally reduced with 15 mM DTT and alkylated with 50 mM iodoacetimide. Samples were diluted 3:1 with 50 mM ammonium bicarbonate prior to overnight digestion with trypsin at 37°C. The digested samples were spun at 14.5 k RPM for 60 min on a benchtop centrifuge to remove particulate matter. The supernatant was collected and desalted with a Michrom (Auburn, CA) C18 peptide trap prior to LC-MS/MS analysis according to the manufactureer's instructions. After desalting the samples were concentrated *in vacuo *to 10 μl.

### LC-MS/MS analysis

4 μl of the desalted peptide mixture diluted to 100 μl was injected onto the column. The peptide mixture was separated on a Symmetry BEH 130 C18 (1.7 μm, 75 μm × 250 mm)column using a Waters UPLC Nano-Acquity system (Milford, MA). The peptides were eluted at a flow of 0.250 μl/min using a linear gradient of acetonitrile with 0.1% formic acid from 2% to 50% over 320 minutes. Eluted peptides were ionized at 1.7 kV and the ions analyzed by a ThermoFisher LTQ-XL mass spectrometer (San Jose, CA). In the ion trap, the top 10 ions were selected for MS/MS by using a data dependent triple play scheme. Dynamic exclusion was set after two scans to facilitate identification of low abundance proteins.

The acquired data was searched in the Bioworks Browser with the SEQUEST search algorithm. Due to the limited size of the rabbit database, proteins from human, rat and rabbit from the NCBI non-redundant database were selected as the database. In order for a protein to be considered, the protein must have a probability of at least 0.001 in SEQUEST with at least two unique peptides with a peptide probability of 0.01. In addition, the peptide must have Xcorr scores above default values set by Bioworks (+1 = 1.5, +2 = 2.0, +3 = 2.5, +4 = 3.0). Proteins that met all of these requirements were determined to be selected as sufficient to be included as identified using MS. After identification, a literature search was performed to determine the function and location of the protein in the cell.

### Immunohistochemistry

Previously fixed and mounted corneal slices were placed onto heating blocks at 55°C for ten minutes to melt the paraffin. Slides were deparaffinized in 2 washes of xylenes for 10 minutes. Slides were rinsed twice for 2 minutes in 100% alcohols (18:1:1, 100% ethanol:100% methanol:100% isopropanol) followed by two 2 minute rinses in a 95% solution of the 100% alcohols. Slides were placed in an 80% solution of the 100% alcohols for 2 minutes followed by 5–6 washes with deionized water for 2 minutes each. Slides were incubated in blocking solution that consisted of 10% normal bovine (for osteoglycin staining) or donkey (for gamma catenin and perlecan stainings) serum (Jackson Immunoresearch Laboratories, West Grove, PA, USA) in Tris Buffered Saline Buffer (TBS; 100 mM Tris pH 7.4, 138 mM NaCl, 27 mM KCl) overnight at +4°C. Antibody incubations were performed in TBSN supplemented with 10% of respective normal serum. Dilutions (1:100)were used for all primary and secondary antibodies. Incubation times were 2 hours with primary antibody and 1 hour with secondary antibody at room temperature on an orbital shaker at slow speed. Samples were washed 5× for 5 min. each in TBS containing 0.1% NonIdet P40 (TBSN) after antibody incubations. Primary antibodies sc-7900, sc-25848 or sc-47277 (Santa Cruz Biotechnology, Santa Cruz, CA, USA) were used to stain against gamma catenin, perlecan or osteoglycin, respectively. Donkey anti-rabbit, FITC-labeled antibody or bovine anti-goat, TRITC-labeled antibody (Jackson Immunoresearch Laboratories, West Grove, PA, USA) were used to stain against gamma catenin, perlecan or osteoglycin, respectively. After the final wash, slides were quickly rinsed twice in TBS and dried at room temperature. A drop of ProLong Gold antifade reagent with DAPI (Invitrogen, Carlsbad, CA, USA) was applied onto the slides. Slides were covered with cover slips. Images were taken using BD Pathway 435 confocal bioimager (BD BioSciences, Rockville, MD, USA).

## Competing interests

MM and JS have no competing interests. PS has been employed by ThermoFisher within the past five years.

## Authors' contributions

MM was responsible for the sample preparation, mass spectrometric analysis and data analysis. PS was responsible for the corneal tissue staining. MM and PS drafted the manuscript. JS is the principal investigator, supervised the project, and provided final additions and edits to this manuscript.

## Supplementary Material

Additional file 1 **Protein List of all proteins identified in SEQUEST analysis**. additional table. Click here for file
